# Rural health workers and their work environment: the role of inter-personal factors on job satisfaction of nurses in rural Papua New Guinea

**DOI:** 10.1186/1472-6963-12-156

**Published:** 2012-06-12

**Authors:** Rohan Jayasuriya, Maxine Whittaker, Grace Halim, Tim Matineau

**Affiliations:** 1 School of Public Health and Community Medicine, University of New South Wales, Sydney, Australia; 2 School of Population Health, University of Queensland, Brisbane, Australia; 3 Liverpool School of Hygiene and Tropical Medicine, Liverpool, United Kingdom

## Abstract

**Background:**

Job satisfaction is an important focal attitude towards work. Understanding factors that relate to job satisfaction allows interventions to be developed to enhance work performance. Most research on job satisfaction among nurses has been conducted in acute care settings in industrialized countries. Factors that relate to rural nurses are different. This study examined inter-personal, intra-personal and extra-personal factors that influence job satisfaction among rural primary care nurses in a Low and Middle Income country (LMIC), Papua New Guinea.

**Methods:**

Data was collected using self administered questionnaire from rural nurses attending a training program from 15 of the 20 provinces. Results of a total of 344 nurses were available for analysis. A measure of overall job satisfaction and measures for facets of job satisfaction was developed in the study based on literature and a qualitative study. Multi-variate analysis was used to test prediction models.

**Results:**

There was significant difference in the level of job satisfaction by age and years in the profession. Higher levels of overall job satisfaction and intrinsic satisfaction were seen in nurses employed by Church facilities compared to government facilities (P <0.01). Ownership of facility, work climate, supervisory support and community support predicted 35% (R2 =0.35) of the variation in job satisfaction. The factors contributing most were work climate (17%) and supervisory support (10%). None of these factors were predictive of an intention to leave.

**Conclusions:**

This study provides empirical evidence that inter-personal relationships: work climate and supportive supervision are the most important influences of job satisfaction for rural nurses in a LMIC. These findings highlight that the provision of a conducive environment requires attention to human relations aspects. For PNG this is very important as this critical cadre provide the frontline of primary health care for more than 70% of the population of the country. Many LMIC are focusing on rural health, with most of the attention given to aspects of workforce numbers and distribution. Much less attention is given to improving the aspects of the working environment that enhances intrinsic satisfaction and work climate for rural health workers who are currently in place if they are to be satisfied in their job and productive.

## Background

Employees have views about aspects of their job, their career and for whom they work. Job satisfaction is an emotional reaction to an employee's work situation. The ‘most focal employee attitude is job satisfaction’ [1]. Locke [2] defined job satisfaction as “…a pleasurable or positive emotional state resulting from the appraisal of one’s job or job experiences’. There are broad differences in what people expect from their jobs and how they react. The relationship between job satisfaction and job performance has been studied for a long time. Saari and Judge [1] in a review of studies stated “it does appear job satisfaction is, in fact a predictor of performance, and the relationship is even stronger for professional jobs”. The overall impression about one's job in terms of specific aspects of the job (e.g., compensation, autonomy, colleagues) can be connected with specific results, such as productivity.

Herzberg [3] developed a two-factor theory of job satisfaction: "motivation" and "hygiene". According to Herzberg's theory, if handled properly, hygiene issues cannot motivate workers but can minimize dissatisfaction. Hygiene factors include organizational policies, supervision, salary, interpersonal relations and working conditions. They are variables related to the worker's environment. A worker's job satisfaction is also influenced by situational factors associated with the work itself, labelled intrinsic satisfaction. These include outcomes directly derived from work such as the nature of their jobs, achievement in the work, promotion opportunities, and chances for personal growth and recognition. Because such factors were associated with high levels of job satisfaction, Herzberg referred to them as "motivation factors".

There has been considerable research on job satisfaction in nursing. The early studies were synthesized in meta-analyses by Blegen [4] and Irvine and Evans [5] and indicated that the factors with the strongest relationship to job satisfaction were related to work and environment. The employment climate for nurses in the 1990s was different to the present and changes have taken place due to nurses having intentions to leave for further education and pay [6]. There have been, many reviews of literature on job satisfaction of nurses since, including studies in acute hospital settings in industrialised countries [6-8] with the most recent by Hayes et al. [9]. Few studies have considered job satisfaction of nurses working in rural areas in Australia [10], Canada [11] and South Africa [12]. Factors that relate to job satisfaction of rural nurses are diverse and different [11].

There have only been a handful of published studies that have researched job satisfaction among health workers in Low and Middle Income countries (LMIC). Studies have examined nurses working in hospitals in South Africa [13,14], community health workers in Iran [15] and health personnel including nurses in Turkey [16]. Only a very few have studied aspects of job satisfaction of nurses who work in Primary Health Care and in rural settings in LMIC [12,17,18]. A limitation of these studies is that they have been conducted in a single organization [18] or in one district of the country [12,17] and the findings cannot be generalised more widely.

In a recent review of job satisfaction, Hayes et al. [9] conceptualized that factors related to job satisfaction can be categorised to three groups: Intra-personal factors, “those characteristics of the nurse”; Inter-personal factors, as those that relate to the interaction of the person to others and Extra-personal factors, that are beyond the interaction with others and “are influenced by institutions or government policies”. We use this classification in the review of factors.

The aim of this study is to examine intra-personal, inter-personal and extra-personal factors that relate to job satisfaction among rural primary care nurses in a LMIC, Papua New Guinea. The secondary aim is to examine how job satisfaction influences their intention to leave. We review the literature on factors that relate to job satisfaction in nurses, with a focus on rural settings. The methods in the study are presented, followed by the findings. Finally, we discuss the findings in relation to current evidence on factors that influence job satisfaction of nurses and in the context of its practical implications for personnel management, productivity and efficiency of health services in LMIC countries.

## Review of literature on factors relating to job satisfaction in nurses

The classification by Hayes et al. [9] is used in presenting the review of literature. The review also includes findings from a qualitative study of rural health workers in PNG [19], which provided insight to relationships and contributed to the development of measures used in this study.

### Intrapersonal factors

The literature from industrialized countries differentiates between three groups of nurses by age, baby boomers (born between 1945 and 65); generation X (born between 1965 and 1979) and generation Y (1980 onwards). Wilson [20] found that baby boomers were more satisfied with pay and scheduling and Lavoie-Tremblay [21] found that young nurses belonging to generation Y had different attitudes and values to work. Longer educational preparedness (years of education) have been attributed to nurses having increased critical thinking skills and the need for autonomy, which may lead to dissatisfaction in some environments [22]. These attributes in younger nurses may explain the difference in attitude of generation Y nurses to that of other groups. Working for a longer time in the profession and in a specific unit/hospital was found to lead to higher levels of job satisfaction in some settings [23]. However this relationship was not seen in all settings such as in studies on rural nurses in Canada [11] and South Africa [12].

### Inter-personal factors

Hayes et al. [9] included factors that influence “intrinsic satisfaction”, such as autonomy and providing direct client services and other factors that relate to the inter-relationship of staff in a work setting, such as professional relationships and leadership under the broad category of inter-personal factors. The literature from industrial and organizational psychology makes a distinction between “psychological climate “, “work or organizational climate” and “organizational culture”. The psychological climate “refers to the meanings that people impute to their jobs, co-workers, leaders, pay performance expectations etc.…” [24]. This is measured at an individual level. The collective perceptions of the work environment are defined as organizational climate [24]. Individual perceptions can be shared and can be aggregated to characterize the work unit and are labelled organizational climate. Organizational culture relates to organizational strategy, arrangements and environment that affect the context and milieu within which work groups operate. It is defined as the normative beliefs (values) and shared behavioural expectation (norms) in an organization [25]. The distinction here is that, the climate reflects an individual’s orientation and is a property of the individual, whereas culture reflects systems values and norms and is the property of the system. We use the term work climate to refer to the organizational climate of the work place.

Meta analytic studies have shown the strong relationship between perceptions of work climate and work outcomes, and that this is mediated by work attitudes (including job satisfaction) [26]. Work climate has been related to work performance and mediates the link between human resource management and organizational performance [27]. In studies of nursing work, a positive work climate creates an environment conducive to the development of trust and empowerment, which in turn leads to high quality patient care [28].

Supervisors have a unique relationship with employees. The quality of relationship between the health worker (HW) and their line manager (supervisor) influences the HW’s perceptions of the work climate, either positively or negatively [29].

In a series of studies on work motivation in hospitals, Franco et al. [30] found that work group cooperation and friendliness, teamwork and managerial support and communication were factors that led to worker motivation. Qualitative studies have illustrated how inappropriate behaviour of colleagues in the workplace leads to low HW motivation [31] and that appreciation by colleagues was a significant factor in job satisfaction in rural Vietnam [32].

### Extra-personal factors

In their review of studies, Hayes et al. [9] identified that pay (remuneration) and organizational policies are factors that influence job satisfaction of nurses. A study of primary health care (PHC) nurses in one state in South Africa similarly illustrated the relationship of job dissatisfaction with nurses’ pay and benefits [12]. The measure of pay, had the highest correlation with job satisfaction (r = 0.57, P < 0.001), but was not a predictor of intent to leave [12]. Nurses working in PHC facilities in another state in South Africa, listed poor remuneration, fifth (9.1% of the sample) in a list of determinants for job dissatisfaction, behind workload (28.7%), chronic lack of resources for work (15.8%) and lack of recognition or appreciation (14.9%) [18]. Despite findings that ‘non monetary factors’ motivate health workers in LMIC [33], qualitative and quantitative findings from non physician clinicians in Tanzania, point to the need for salary requirements to be satisfied [34].

Many of the studies of rural nurses have as their objective to investigate rural nurses’ intention to stay (turnover intent) in rural posts. Studies in the 1990s, found the relationship between job satisfaction and intent to stay in rural practice [35,36]. In both studies the respondents were nurses working in community hospitals. In a review of the rural nursing workforce in Canada, Australia and the United States, Bushy et al. [37] noted that ‘rural lifestyle’ affected nurses in all three countries. In a study of public health nurses in rural Canada a measure of community satisfaction was found to relate to job satisfaction [38] . However, neither job satisfaction nor community satisfaction were related to plans to leave in two years. The authors stated that “filter factors” such as demographics (age), personal circumstances (financial needs) and opportunity (availability of employment for spouse) promote or limit retention in rural practice [38]. The limitations of this study were the small sample size, limited analysis and being restricted to one state.

In a nationally representative study of rural nurses in Canada, Kulig et al. [39] were able to confirm the link between satisfaction of one’s home and/or work community and job satisfaction. Based on data from the same study Stewart et al. [40] explored predictors of intent to leave (ITL) a nursing position in rural and remote practice in Canada. Lower community satisfaction and greater dissatisfaction with job scheduling and autonomy at the workplace were significant predictors of ITL within next 12 months. In a more recent study of PHC nurses working in rural south Africa it was found that younger age, higher levels of education, and job satisfaction (measured as a composite index) were predictors of ITL [12]. When specific facets of job satisfaction were considered, supervision was found to be an important factor for ITL.

## Studies in Papua New Guinea

There have been a few studies on the health workforce in Papua New Guinea, especially in rural health services. Davy [41] identified the ambiguity of health workers’ roles in practice despite clearly defined roles and responsibilities in planning and standards documents of the country. A common finding was that Community Health Workers (CHWs) are the primary care providers in the lowest level of health facility and at higher levels are trained to assist nurses. They are most likely to take on roles and responsibilities beyond their training. This concern about not being competent to undertake the work required was a theme for nearly all of the respondents (CHWs, nurses and Health Extension Officers) in the study [41]. In another study of nursing values, most interviewees were of the view that tertiary education for nursing officers had resulted in nurses being able to provide more complex services to patients at hospitals and rural health centres (HCs). A consequence of this is that in many settings, particularly in rural HCs, nurses had progressively assumed responsibility for work that has been traditionally undertaken by medical officers [42].

In our qualitative study in rural PNG, we found that discrepancies in salary between church and government facilities was a de-motivating factor for all health workers (HW). However the limited employment opportunities in the country resulted in their continuing to work in rural areas [19]. In addition the study found that many female health workers were sole income earners and this placed pressure upon them to continue working despite dissatisfaction. Location of housing in close proximity to health facility had mixed blessings, making it easier for them to provide care after-hours and to get to work but often creating work-family conflict as patients have access to them after hours [19].

## Background and context for the study

The population of PNG, of approximately 6.2 million belong to more than 800 language groups spread across 20 provinces. Over 85 percent live in rural areas and as 50 per cent of the total land area is mountainous many rural areas of the country are still inaccessible by road. Difficult terrain including in coastal provinces large sea distances, combined with poor infrastructure and lack of transport, reduces considerably the physical accessibility of health services. PNG’s national government health system is based on a network of 2400 aid posts (many were closed in 2003 due to lack of staff and supplies), 500 health centres, 19 provincial hospitals, a national hospital, and 45 urban clinics [43]. There are limited numbers of private health facilities.

Health services at health centres and sub-centres, rural hospitals and aid posts are known collectively as ‘rural health services’ in PNG. The government is the largest provider of health care, although the church health services operates 45 percent of all rural health centres and sub-centres and employs 23 percent of HW in PNG [44]. Churches are primarily involved in the provision of service to the rural areas and the training of HWs. Government is the main source of finance for both, meeting over 80 per cent of the costs of church health services. Churches provide services to populations in some of the most difficult-to-reach areas of the country. The churches are also responsible for training many of PNG’s health workers, including nurses (6 of the 8 training schools) and CHWs (all of the CHW training schools). The main groups of HW are: CHWs (35%), Nurses (26%), Health Extension Officers (HEOs) (4%) and Medical Officers (3%). Nurses and CHWs form the backbone of primary health care services in rural areas.

It was estimated that in 2000, PNG had a health staff to population ratio of 0.58 HWs (doctors, nurses, HEOs and CHWs) per 1000, considerably lower than the minimum recommended by WHO of 2.28 doctors, nurses and midwives per 1000 in order to achieve the MDGs [45]. With the high population growth rate of 2.7 per annum, there needs to be an increase of 50 per cent of doctors, nurses and HEOs to even maintain this present inadequate ratio [46]. Therefore ensuring the productivity of the workforce that is in place is critical in trying to achieve essential health outcomes from the health system.

## Methods

This paper is based on survey data collected for a study examining rural health worker motivation and performance in Papua New Guinea. The survey was preceded by a qualitative study in three selected districts from three provinces in PNG. Health centres studied were purposively selected to ensure government, private sector and church run facilities were included. Thirty three HWs working in rural health centres were included. Participants were selected to ensure maximum variation in terms of geographical location, health worker category, number of years in service and gender. The in depth interviews and observations were conducted by experienced qualitative researchers from the University of New South Wales and four trained Papua New Guineans. Participants were interviewed on a wide range of factors : their motivation to work, the nature of work, relationships at work and job satisfaction [19]. Findings from the qualitative study were considered in developing the survey instruments (see section on measures).

Data collection for the quantitative study took place in 15 of the 20 provinces in PNG. The data was collected when rural HWs attended a national competency training provided to health workers for the introduction of a new malaria diagnosis and treatment protocol, during 2010 over a six month period. Training of provincial level trainers in the administration of the survey was carried out at one site by members of the research team. The administration of the survey was mainly carried out by the provincial trainers, in some cases with members of the research team in attendance, at provincial and district level. Rural HWs attending the training were provided information about this survey by their trainers and on providing consent to participate were given a self administered survey in English. The anonymity of respondents was ensured as names and other identifiable information were not collected. The completed questionnaires were placed in a sealed envelope by the respondents, collectively packaged by the provincial trainer and posted to the researchers at University of New South Wales (UNSW). Ethical clearance was obtained from the Medical Research Advisory Committee of PNG and the Human Research Ethics Committee at the UNSW.

## Survey instrument development and measures

The survey instrument included demographic and professional information regarding their qualification/s and postings. A series of tested and purpose developed scales to capture constructs related to HW motivation and performance were included in the survey instrument. Tested scales were not available for constructs such as “supervisory support” and “community support” that were relevant for the context. Therefore we developed new scales based on literature and findings from the qualitative study. In other cases we selected items from existing scales and adapted them to suit the context of PNG. A pilot study was carried out at two health centres, to test the content validity and refine survey administration protocols. Only minor changes to the wording in a few questions were required.

Measure of Job satisfaction: The survey measure of job satisfaction developed for this study captures four facets considered in the literature as important for rural health workers. Items were sought from previous studies to measure “satisfaction with supervision”, “satisfaction with co-workers”, “intrinsic job satisfaction” (includes items on the job being “challenging and interesting”, “contact with patients” and “accomplishment”) and “extrinsic job satisfaction” (includes items on remuneration, physical environment and training opportunities). Satisfaction was measured by means of six point Likert scale ranging from 1 (no, I strongly disagree) to 6 (yes, I strongly agree). An “overall job satisfaction” score was calculated by summation of all four facets. The reliability of the measure was estimated by examining internal consistency reliability using Cronbach’s alpha. The Cronbach’s alpha values for this study are (a) satisfaction with supervision = 0.61; (b) satisfaction with co-workers = 0.64; (c) intrinsic job satisfaction = 0.60; (d) extrinsic job satisfaction = 0.63; (e) overall job satisfaction =0.75.

Measure of Work Group Climate: A measure of work group climate developed by Perry et al., [47] which has been tested in many LMIC countries and found to be reliable (Cronbach alpha = 0.81) and valid was used. The Cronbach’s alpha value for this study was 0.73.

Measure of supervisory support: A measure that asked respondents to evaluate their immediate supervisor was used for this study. In most cases, in the health centres respondents identified the immediate supervisor as the officer in charge or sister in charge. A six item measure of supervisory support was developed for this study based on literature and our qualitative findings. An example of an item in the scale is “My supervisor is available for me to talk to when I need to or want to”. A six point Likert scale ranging from 1 (never) to 6 (always) was used. The Cronbach’s alpha value for this study was 0.65.

Measure of the external environment: A four item measure that captured community support was developed from our qualitative work. An example of an item in the scale is “The community supports us to feel safe and secure in this location”. A six point Likert scale ranging from 1 (no, I strongly disagree) to 6 (yes, I strongly agree) was used. The Cronbach’s alpha value for this study was 0.71.

Turnover intention was measured as a single item “I sometimes think of finding another job in another place/area but in the same profession”. The wording of the measure reflected the very tight job market for HWs in rural PNG. The main purpose was to find out intentions of leaving a workplace, but to exclude those who were leaving on retirement and to not work.

Details of the items can be obtained from the corresponding author.

## Data management and analysis

The database for the study contained 1,302 questionnaires from HW in PNG. HW in the study included HEOs, Nurses, CHW and some staff from provincial offices. During data cleaning, questionnaires that had extensive missing values for variables and questionnaires from CHWs who worked at aid posts were excluded. This resulted in 1,282 completed questionnaires for analysis, of which a total of 489 were from nurses. For this study, respondents who had identified their profession as nurse, and worked in rural hospitals (RH), health centre (HC), rural health centre (RHC) and sub health centre (SHC) were included. Nurses who worked in district hospitals or urban health centre were excluded from the analysis. This resulted in a total of 344 nurses being included in the analysis.

Survey results were analysed using PASW 18. Descriptive and bivariate analyses were used to analyse relationships between the main variables of interest. Mean differences were examined using *t*-test and ANOVA for relevant subgroups. Correlations were assessed using Pearson correlation. Prediction models were built for job satisfaction and intention to leave, using sequential regression analysis.

## Results

The majority of respondents were female (70.6%), above 35 years old (55%), married (81%), and had reached an educational level of grade 10–12 before commencing their training as health professionals (82%). Most respondents had six or more years of nursing experience (44%). Housing was provided to 68% and 57% lived in the same compound as the health facility. Fifty seven percent of respondents worked in government facilities and 40% worked in church owned facilities, only approximately 3% of respondents worked in the private sector. Respondents were from 15 provinces in PNG, and the distribution of respondents by region was Islands (137), Southern (48), Highland (58) and Momase (101).

Job satisfaction was measured as both a facet score and a composite score (Table 1). There was significant difference in satisfaction with supervision by age (P < 0.05) and years in the profession (P < 0.05), with those who were older (>35 years) and longer in the profession (>20 years) having higher levels of satisfaction. There was no significant difference in measures of satisfaction with co-workers and intrinsic satisfaction, with demographic variables. When the partner of the respondent did not work, there was higher level of extrinsic satisfaction (e.g. remuneration) (P < 0.05). Overall job satisfaction (measured with the composite score) showed significant difference by years in profession (P < 0.01), partner not at work (P < 0.05) and housing being provided (P < 0.05).

**Table 1:** Facets of job satisfaction, overall job satisfaction and retention by demographics

	**n (%)**	**Satisfaction supervision**	**Satisfaction co-workers**	**Intrinsic satisfaction**	**Extrinsic satisfaction**	**Overall Satisfaction**	**Retention**
Gender (n = 343)							
Female	242 (71%)	3.72	4.60	4.98	3.47	4.14	4.08
Male	101 (29%)	3.76	4.69	4.82	3.32	4.05	4.27
Age (n = 329)							
<=35	140 (41%)	3.51^a^	4.61	4.91	3.32	4.03	4.54^c^
>35	189 (55%)	3.89	4.65	4.96	3.54	4.19	3.83
Years in Profession (n = 338)							
0-5	63 (18%)	3.59^b^	4.72	5.00	3.44	4.14^b^	4.53^c^
6-20	151 (44%)	3.52	4.57	4.81	3.27	3.96	4.41
>20	124 (36%)	4.04	4.65	5.02	3.61	4.27	3.60
Level of Education (n = 341)							
Grade 1-9	58 (17%)	4.05	4.78	4.93	3.66	4.32	3.20^c^
Grade 10-12	283 (82%)	3.67	4.60	4.92	3.38	4.07	4.31
Marital status (n = 342)							
Married	277 (81%)	3.76	4.62	4.90	3.42	4.10	4.14
Single	33 (10%)	3.56	4.77	5.14	3.70	4.24	4.61
Widowed/separated/divorced	32 (9%)	3.62	4.50	4.93	3.13	4.01	3.55
Distance from work (n = 339)							
In same compound	197 (57%)	3.71	4.65	5.00	3.52	4.17	4.22
Less than 30 minutes walk	70 (20%)	3.77	4.61	4.88	3.40	4.11	4.09
More than 30 minutes travel time	72 (21%)	3.76	4.61	4.86	3.26	4.00	4.00
Partner work (n = 280)							
Yes	150 (44%)	3.68	4.54	4.81	3.25^a^	3.99^a^	4.42^b^
No	130 (38%)	3.84	4.67	4.98	3.60	4.21	3.75
Housing provided (n = 343)							
Yes	233 (68%)	3.74	4.68	4.98	3.48	4.16^a^	4.11
No	110 (32%)	3.67	4.51	4.80	3.28	3.97	4.18
Ownership (n = 342)							
Government	196 (57%)	3.68	4.55	4.88	3.28	4.00^z^	4.13
Church	136 (40%)	3.84	4.75	5.01	3.63	4.26	4.16
Private/Others	10 (3%)	3.10	4.83	5.05	3.53	4.19	4.20

^a^ = p value < 0.05; ^b^ = p value < 0.01; ^c^ = p value < 0.001.

Younger nurses (<35 years) and those with less years in the profession (0–5 years) had significantly higher agreement with turnover intention (P < 0.001). A higher level of turnover was expressed by nurses whose partners had work (P < 0.05). There was no significant difference in turnover intention between nurses in Government, Church and private sectors.

There was no difference in job satisfaction by each facet or composite score between nurses in different regions (Table 2). However, a significant higher level of extrinsic satisfaction and overall job satisfaction was seen in by ownership of facility with Church HWs having higher values for extrinsic satisfaction than HWs in Government facilities (P < 0.01). The work climate was significantly different by region with the highest being in the Highlands region followed by the Islands region. Work climate was significantly higher (P < 0.001) in Church owned facilities and a higher level of community support was found for nurses in Church facilities over those in government facilities (P < 0.05).

**Table 2:** Association of facets of Job satisfaction, factors and retention with region and facility ownership

	**Region**				**p value**	**Ownership**		**p value**
	**1**	**2**	**3**	**4**		Government*	Church*	
Satisfaction supervision	3.75	3.70	3.70	3.77	0.984	3.68	3.84	0.296
Satisfaction co-workers	4.62	4.50	4.79	4.70	0.239	4.55	4.75	0.073
Intrinsic satisfaction	4.84	4.91	4.85	5.04	0.414	4.88	5.01	0.148
Extrinsic satisfaction	3.63	3.51	3.41	3.23	0.222	3.28	3.63	**0.010**
Overall job satisfaction	0.30	0.29	0.28	0.27	0.959	0.27	0.30	**0.003**
Supervision	3.45	3.23	3.28	3.64	0.103	3.30	3.52	0.132
Work Climate	4.93	4.54	5.05	4.61	**0.000**	4.57	4.88	**0.000**
Community support	14.00	13.19	13.84	13.85	0.301	13.26	14.19	**0.013**
Retention	3.67	4.13	4.28	4.27	0.239	4.13	4.16	0.895

*after excluding private + others.

Relationship of work climate to type of facility was tested using an ANOVA. A significant difference in work climate was found between facilities [F (3, 312) = 3.85; P <0.01] with lower scores for health centres (HC, mean 36.34) than rural health centres (RHC, mean 38.56) and sub health centres (SHC, mean 38.65) as shown by post-hoc Tukey test.

As expected there was a significant negative association between overall job satisfaction and turn over intention (r = −0.18, p <0.05). High correlations were found between job satisfaction and supervision (r = 0.49), work climate (r = 0.44) and community support (r = 0.33) (Table 3).

**Table 3:** Correlation between variables

		**1**	**2**	**3**	**4**	**5**	**6**	**7**	**8**	**9**	**10**	**11**	**12**
1	Age	1											
2	Years of profession	0.882^c^	1										
3	Years working in the health centre	0.579^c^	0.581^c^	1									
4	Satisfaction supervision	0.147^b^	0.181^b^	0.091	1								
5	Satisfaction co-workers	−0.015	0.000	0.044	0.234^c^	1							
6	Intrinsic satisfaction	0.040	0.028	0.009	0.395^c^	0.385^c^	1						
7	Extrinsic satisfaction	0.099	0.112^a^	0.135^a^	0.444^c^	0.120^c^	0.305^c^	1					
8	Overall job satisfaction	0.118^a^	0.130^a^	0.119^a^	0.758^c^	0.493^c^	0.653^c^	0.781^c^	1				
9	Retention	−0.275^c^	−0.271^c^	−0.122^a^	−0.162^b^	−0.010	−0.016	−0.219^c^	−0.180^b^	1			
10	Supervision	0.062	0.063	0.065	0.592^c^	0.196^c^	0.304^c^	0.225^c^	0.485^c^	−0.106	1		
11	Work Climate	0.000	0.019	−0.004	0.338^c^	0.440^c^	0.381^c^	0.167^b^	0.440^c^	−0.126^a^	0.334^c^	1	
12	Community Support	0.052	0.052	0.031	0.274^c^	0.169^b^	0.259^c^	0.243^c^	0.329^c^	−0.113^a^	0.244^c^	0.321^c^	1

^a^ = p value < 0.05; ^b^ = p value < 0.01; ^c^ = p value < 0.001.

Using stepwise regression analysis a model for predicting rural nurse job satisfaction was developed (Table 4). In the final model, ownership, work climate, supervisory support, and community support were significant predictors. They account for 35% of the total variance in job satisfaction. Age, education level, number of years in the profession, and region were not significant predictors of job satisfaction. The highest change in job satisfaction (based on R^2^) was obtained with the inclusion of work climate (17%) followed by supervision (10%).

**Table 4:** Predictors of Job Satisfaction

**Predictor**	**B**	**R**^**2**^	**R**^**2**^**change**	**B**	**SE B**	**t**	**P value**
Age	0.117	0.014	0.014	0.114	0.057	2.00	0.05
Years in profession	0.133	0.018	0.004	0.031	0.130	0.24	0.81
Spouse work	0.229	0.052	0.035	1.808	1.221	1.48	0.14
Education	0.248	0.061	0.009	−2.574	2.232	−1.15	0.25
Region	0.268	0.072	0.011			0.82	0.48
Health centre ownership	0.316	0.100	0.028	3.368	1.299	2.59	0.01
Work Climate	0.520	0.270	0.170	0.740	0.108	6.88	0.00
Supervisory support	0.614	0.377	0.107	0.430	0.076	5.66	0.00
Community support	0.624	0.390	0.013	0.374	0.180	2.07	0.04
Total R^2^ = 0.390							
Total adjusted R^2^ = 0.353							

In comparison the predictors of intention to turn over were very different (Table 5). The only significant predictors, in the presence of other factors were age and “spouse’s work”. If the spouse was working, this had a positive association with intention to turn over. None of the external factors at work or job satisfaction were significant predictor in multivariate analysis, though they were associated in univariate analysis. The factors were only able to predict only 7% of variance, with age contributing for most of the variation.

**Table 5:** Predictors of Intention to leave

**Predictor**	**B**	**R**^**2**^	**R**^**2**^**change**	**B**	**SE B**	**t**	**P value**
Age	0.275	0.076	0.076	−0.050	0.010	−5.14	0.00
Years in profession	0.279	0.078	0.002	−0.020	0.019	−1.05	0.30
Spouse work	0.287	0.082	0.004	−0.524	0.213	−2.46	0.02
Education	0.301	0.090	0.008	0.672	0.359	1.87	0.06
Region	0.307	0.094	0.004			0.37	0.77
Health centre ownership	0.318	0.101	0.007	−0.057	0.232	−0.25	0.81
Job Satisfaction	0.335	0.112	0.011	−0.019	0.013	−1.47	0.14
Supervisory support	0.336	0.113	0.001	−0.005	0.018	−0.31	0.76
Work Climate	0.357	0.128	0.016	−0.020	0.025	−0.8	0.43
Community support	0.358	0.128	0.016	−0.033	0.040	−0.82	0.42
Total R^2^ = 0.128							
Total adjusted R^2^ = 0.071							

## Discussion

This study contributes to the limited literature on job satisfaction of nurses working in LMIC. By examining nurses working in primary care units in rural areas in a country with difficulties of access and isolation, this study provides unique insight to whether factors common to settings in developed countries prevail. The study included nurses from most provinces and regions in PNG and therefore is representative of rural nurses in the country.

The most significant predictors of job satisfaction of rural nurses in PNG were work climate and supervision. The relationship of work climate to motivation and satisfaction has been examined in studies in developed countries [10,38,48]. Sellgren et al. [49] defined work climate as the way people perceive their work environment and found a significantly correlation to job satisfaction. Relationships with colleagues, one of the facets of job satisfaction studied, was found to be the strongest predictor in one study in a hospital setting [50]. However, in another study, only 20% identified lack of teamwork and collegial support to be an issue [51].

Studies in LMIC have not used valid measures to examine work climate. For instance, Hagopian [52] used a single item “hospital is a fun place to work”, to capture work climate. Most of the evidence comes from qualitative studies that have identified the importance of inter-personal relationships in the workplace in LMIC as factors for HW motivation [17,31,32]. In this study, we used a validated measure [47] and provide the first evidence of the strong relationship of work climate with job satisfaction in rural settings of a LMIC.

In a context where HWs work and live in close proximity to their fellow workers in rather isolated circumstances, good interpersonal relations are vital to job satisfaction. In our analyses, we found that the level of work climate in church owned facilities was significantly higher than in government facilities, providing some evidence that certain aspects of the “climate” in faith-based organizations may have an impact on the job satisfaction of staff. However, the study did not use a validated measure of organizational culture, therefore further studies are needed to tease out how the work climate in faith based organizations influences HW motivation and job satisfaction and which aspects of organizational culture influence health workers.

A difference in the level of work climate by type of facility was found in this study. Of the four types of facilities, it was lowest in the HCs. The work climate was significantly lower in health centres in comparison with RHCs and SHCs. As the numbers of staff at health centres are in general higher than these two facilities, it may mean that less positive inter-personal relationships are developed at larger facilities. However, this needs further investigation.

The relationship of supervisory support to nurses’ job satisfaction in hospital settings in developed countries is well established [9]. Much of the qualitative literature from LMIC discusses the importance of supervision for motivation and performance of staff, especially in primary care settings [33,53]. Lack of supportive supervision has been shown to negatively affect HW and decrease their motivation to work and also their attitude towards clients [17]. Among hospital staff, supervision was a factor that contributed to job satisfaction in rural South Africa [12]. In this study, we measured supervisory support with a reliable scale and found that it was significant predictor of job satisfaction. The findings of the study show that there is significant difference in supervisory support provided by immediate supervisors in a facility and that this has a significant impact on job satisfaction of nurses.

Penz et al. [11] state “the concept of community satisfaction is a relatively unique aspect… for nurses in rural populations”. The measure of community support used in the study was not the same as community satisfaction measured in studies in Canada [38-40]. Future studies may need to explore this aspect.

An important aspect of community relations are the perceptions the nurses (who are mostly females) have of physical security. In our study the measure of community support included items that relate to “safety’ and the role that the community plays in securing safety for HW in PNG. Others have mentioned issues of safety in rural settings in LMIC, but have not measured it and provided empirical evidence [33]. Issues of security were mentioned in our qualitative component of the larger study, and the surveys confirm these findings.

Pay and work conditions are most frequently cited as affecting job satisfaction in studies in hospitals [12,14]. Although nurses are frustrated with such external factors, there is much evidence that the work of nursing itself continues to be a strong job satisfier. This is an aspect seen in qualitative studies in LMIC [33]. We found empirical evidence of the positive relationship of intrinsic satisfaction with job satisfaction. Our qualitative work [19], gave insight to the satisfaction nurses have of being able to carry out clinical work that was directly seen to benefit patients. This aspect of job autonomy, has also been mentioned in the literature [49]. Further studies are required to ascertain the effects of job enlargement, on motivation and job satisfaction in such settings in LMIC.

Intention to turnover has been a topic of much of the research on nursing workforce in developed countries with most studies conducted in urban hospital settings. Of the few studies in rural and remote areas in developed countries, a link between job satisfaction and community satisfaction with intention to leave has been shown [40]. Job satisfaction was not a predictor of rural nurses intention to leave in PNG, despite a low but significant correlation between the varaibles. This seems similar to the findings of public health nurses in Canada, where “filter factors” seem to be more important [38]. In fact, the only significant predictors were age and “spouse work”. This concurs with the observations from nursing studies in developed countries that nurses belonging to “generation Y” had different attitudes towards intention to stay. When the spouse of the nurse did not find work in the rural area there was higher intention to leave to find a job elsewhere [38].

In our qualitative study it was found that discrepancies in salary between church and government facilities was a demotivating factor, but the limited employment opportunities resulted in their continuing to work in rural areas [19]. In addition the qualitative study found that many female health workers were sole income earners and this placed pressure to continue working. Given this context it was not surprising to find that job satisfaction was not a significant predictor of intention to turnover. Studies of primary health care nurses in rural South Africa, have found that turnover intent is significantly predicted by satisfaction with supervision [12]. Turnover intention has also been linked to satisfaction with co-workers and the work climate [12]. Although the PNG nurses did not state an intention to physically leave, decreased motivation and therefore productivity are a “passive” response to the unsatisfactory work climate.

In PNG senior managers in the provincial and national departments of health need to address some of the implications of the study. The World Health Report [45] identified unmotivated staff as one of the top ten leading causes of inefficiency of a health system. Whilst not denying the absolute need to increase the number and improve the distribution of primary health care workers in PNG to meet the defined national standards [42] the need to ensure a productive workforce, both those in place and those being newly hired is also important. The lag time in “production” of this workforce has been discussed in the PNG Health Workforce Plan [42], Ensuring maximum efficiency from the existing human resource is an important strategy in the short to medium term. The study has identified an important element of this “improved efficiency” namely good supervision supporting a positive work climate. Identifying the barriers to supervision, addressing these, and ensuring that the style of supervision is supportive, thereby enhancing the work climate are important short term strategies, that should be affordable through careful planning.

There are some limitations in our study that need to be considered. While the study used measures based on literature and our own field studies, some measures require further refinement to increase their reliability. A number of measures were developed for this study, that requires further validation research. The findings from the quantitative study have reinforced some of the findings of the qualitative study [19] and vice versa, providing some triangulation to our results. As the primary focus of the study was on HW motivation and performance, more accurate measures of retention (intention to turn over) were not used to reduce the burden on the respondents. While we had good representation from most of the provinces in PNG, it must be taken into account that the survey was administered to HWs who were selected for training on malaria, which may have caused selection bias.

## Conclusions

This study was designed to identify factors that relate to job satisfaction of rural nurses in a LMIC country. The findings indicate that inter-personal factors, such as work climate and supportive supervision were significant contributors to job satisfaction. These findings concur with other recent qualitative studies of the importance of personnel management in rural work places in order to sustain a motivated work force and thereby provide high quality services. Organizational culture (as measured by ownership of facility) and community support were the extra-personal factors of significance.

Papua New Guinea has in its most recent 10 year strategy committed to “focus on where the 87% of our population reside; ...[with] focus on improving service delivery; …it will focus on primary health care” [54]. The rural health strategy of PNG is underpinned by a motivated and productive rural health workforce and a key professional group are nurses. There are current plans to enhance rural health services, by addressing the staffing shortage and improving the run down facilities in rural areas. However, the strategies do not consider important aspects of maintaining a satisfied workforce. The importance of the work climate and supervisory support for job satisfaction of nurses in rural settings in PNG is empirically shown in this study.

This study adds to the meagre literature on health work force issues from LMIC. While some findings are similar to those factors that relate to job satisfaction in other settings, the differences that are a result of the context is shown. Further research is indicated to explore the relationships between job satisfaction of health workers with performance and productivity in rural settings.

## Competing interests

The authors declare that they have no competing interests.

## Authors' contributions

RJ conceived the study, designed the study, co-ordinated data collection, directed data analysis and drafted the manuscript. MW participated in design and helped draft the manuscript. GH carried out data management and data analysis and helped draft the manuscript . TM participated in design and helped draft the manuscript. All authors read and approved the final manuscript.

## Pre-publication history

The pre-publication history for this paper can be accessed here:

http://www.biomedcentral.com/1472-6963/12/156/prepub

## References

[B1] Saari LM Judge TA Employee attitudes and job satisfaction Hum Resour Manage 2004 43 395 407 10.1002/hrm.20032

[B2] Locke E Dunnette MP The Nature and Causes of Job Satisfaction Handbook of Industrial and Organizational Psychology 1976 Rand McNally, Chicago 1297 1350

[B3] Herzberg F Work and the Nature of Man 1966 Crowell, New York

[B4] Blegen MA Nurses' job satisfaction: a meta-analysis of related variables Nurs Res 1993 42 36 41 8424066

[B5] Irvine DM Evans MG Job satisfaction and turnover among nurses: integrating research findings across studies Nurs Res 1995 44 246 253 7624236

[B6] Coomber B Barriball KL Impact of job satisfaction components on intent to leave and turnover for hospital-based nurses: a review of the research literature Int J Nurs Stud 2007 44 297 314 10.1016/j.ijnurstu.2006.02.004 16631760

[B7] Lu H While AE Barriball KL Job satisfaction among nurses: a literature review Int J Nurs Stud 2005 42 211 227 10.1016/j.ijnurstu.2004.09.003 15680619

[B8] Utriainen K Kyngas H Hospital nurses' job satisfaction: a literature review J Nurs Manag 2009 17 1002 1010 10.1111/j.1365-2834.2009.01028.x 19941574

[B9] Hayes B Bonner A Pryor J Factors contributing to nurse job satisfaction in the acute hospital setting: a review of recent literature J Nurs Manag 2010 18 804 814 10.1111/j.1365-2834.2010.01131.x 20946216

[B10] Hegney D McCarthy A Job satisfaction and nurses in rural Australia J Nurs Adm 2000 30 347 350 10.1097/00005110-200007000-00007 10953692

[B11] Penz K Stewart NJ D'Arcy C Morgan D Predictors of job satisfaction for rural acute care registered nurses in Canada West J Nurs Res 2008 30 785 800 10.1177/0193945908319248 18612089

[B12] Delobelle P Rawlinson JL Ntuli S Malatsi I Decock R Depoorter AM Job satisfaction and turnover intent of primary healthcare nurses in rural South Africa: a questionnaire survey J Adv Nurs 2011 67 371 383 10.1111/j.1365-2648.2010.05496.x 21044134

[B13] Pillay R Work satisfaction of professional nurses in South Africa: a comparative analysis of the public and private sectors Hum Resour Health 2009 7 15 10.1186/1478-4491-7-15 19232120 PMC2650673

[B14] Kekana HP du Rand EA van Wyk NC Job satisfaction of registered nurses in a community hospital in the Limpopo Province in South Africa Curationis 2007 30 24 35 17703820 10.4102/curationis.v30i2.1068

[B15] Kebriaei A Moteghedi MS Job satisfaction among community health workers in Zahedan District, Islamic Republic of Iran East Mediterr Health J 2009 15 1156 1163 20214129

[B16] Bodur S Job satisfaction of health care staff employed at health centres in Turkey Occup Med (Lond) 2002 52 353 355 10.1093/occmed/52.6.353 12361997

[B17] Manongi RN Marchant TC Bygbjerg IC Improving motivation among primary health care workers in Tanzania: a health worker perspective Hum Resour Health 2006 4 6 10.1186/1478-4491-4-6 16522213 PMC1450300

[B18] Bester CL Engelbrecht MC Job satisfaction and dissatisfaction of professional nurses in primary health care facilities in the free state province of South Africa Africa Journal of Nursing and Midwifery 2009 11 104 117

[B19] Jayasuriya R Razee H Bretnall L Whittaker M Yap L Chakumai K Voices from the field: Factors Influencing Rural Health Workers Performance in Papua New Guinea 2011 University of New South Wales, Sydney

[B20] Wilson B Squires MAE Widger K Cranley L Tourangeau ANN Job satisfaction among a multigenerational nursing workforce J Nurs Manag 2008 16 716 723 10.1111/j.1365-2834.2008.00874.x 18808466

[B21] Lavoie-Tremblay M Paquet M Duchesne M-A Santo A Gavrancic A Courcy F Gagnon S Retaining Nurses and Other Hospital Workers: An Intergenerational Perspective of the Work Climate J Nurs Scholarsh 2010 42 414 422 10.1111/j.1547-5069.2010.01370.x 21091624

[B22] Zurmehly J The relationship of educational preparation, autonomy, and critical thinking to nursing job satisfaction J Contin Educ Nurs 2008 39 453 460 10.3928/00220124-20081001-10 18990891

[B23] Bjork IT Samdal GB Hansen BS Torstad S Hamilton GA Job satisfaction in a Norwegian population of nurses: a questionnaire survey Int J Nurs Stud 2007 44 747 757 10.1016/j.ijnurstu.2006.01.002 16504197

[B24] James LR Choi CC Ko C-HE McNeil PK Minton MK Wright MA Kim K-i Organizational and psychological climate: A review of theory and research European Journal of Work and Organizational Psychology 2008 17 5 32 10.1080/13594320701662550

[B25] Cooke RA Szumal JL Measuring normative beliefs and shared behavioral expectations in organizations : the reliability and validity of the organizational culture inventory Anglais 1993 72 1299 1330

[B26] Parker CP Baltes BB Young SA Huff JW Altmann RA LaCost HA Roberts JE Relationships between psychological climate perceptions and work outcomes: a meta-analytic review Journal of Organizational Behavior 2003 24 389 416 10.1002/job.198

[B27] Gelade GA Ivery M The impact of human resource management and work climate on organizational performance Personnel Psychology 2003 56 383 404 10.1111/j.1744-6570.2003.tb00155.x

[B28] Laschinger HK Finegan J Shamian J The impact of workplace empowerment, organizational trust on staff nurses' work satisfaction and organizational commitment Health Care Manage Rev 2001 26 7 23 11482178 10.1097/00004010-200107000-00002

[B29] Purcell J Hutchinson S Front-line managers as agents in the HRM-performance causal chain: theory, analysis and evidence Human Resource Management Journal 2007 17 3 20 10.1111/j.1748-8583.2007.00022.x

[B30] Franco LM Bennett S Kanfer R Stubblebine P Determinants and consequences of health worker motivation in hospitals in Jordan and Georgia Soc Sci Med 2004 58 343 355 10.1016/S0277-9536(03)00203-X 14604620

[B31] Lindelow M Serneels P The performance of health workers in Ethiopia: results from qualitative research Soc Sci Med 2006 62 2225 2235 10.1016/j.socscimed.2005.10.015 16309805

[B32] Dieleman M Cuong PV Anh LV Martineau T Identifying factors for job motivation of rural health workers in North Viet Nam Hum Resour Health 2003 1 10 10.1186/1478-4491-1-10 14613527 PMC280735

[B33] Mathauer I Imhoff I Health worker motivation in Africa: the role of non-financial incentives and human resource management tools Hum Resour Health 2006 4 24 10.1186/1478-4491-4-24 16939644 PMC1592506

[B34] Chandler CI Chonya S Mtei F Reyburn H Whitty CJ Motivation, money and respect: a mixed-method study of Tanzanian non-physician clinicians Soc Sci Med 2009 68 2078 2088 10.1016/j.socscimed.2009.03.007 19328607

[B35] Dunkin J Juhl N Stratton T Geller J Ludtke R Job satisfaction and retention of rural community health nurses in North Dakota J Rural Health 1992 8 268 275 10.1111/j.1748-0361.1992.tb00367.x 10122981

[B36] Pan S Dunkin J Muus KJ Harris R Geller JM A logit analysis of the likelihood of leaving rural settings for registered nurses J Rural Health 1995 11 106 113 10.1111/j.1748-0361.1995.tb00403.x 10143271

[B37] Bushy A International perspectives on rural nursing: Australia, Canada, USA Aust J Rural Health 2002 10 104 111 12047505 10.1046/j.1440-1584.2002.00457.x

[B38] Bektus MH MacLeod MLP Retaining Public Health Nurses in British Columbia: the influence of job and community satisfaction Can J Public Health 2004 95 54 58 14768743 10.1007/BF03403635 PMC6975568

[B39] Kulig JC Stewart N Penz K Forbes D Morgan D Emerson P Work setting, Community Attachment and Satisfaction among rural and remote nurses Public Health Nurs 2009 26 430 439 10.1111/j.1525-1446.2009.00801.x 19706126

[B40] Stewart NJ D’Arcy C Kosteniul J Andrews ME Morgan D Forbes D MacLeod MLP Kulig JC Pitblado R Moving on? Predictors of intent to leave among rural and remote RNs in Canada J Rural Health 2011 27 103 113 10.1111/j.1748-0361.2010.00308.x 21204977

[B41] Davy C Contributing to the wellbeing of primary health care workers in PNG J Health Organ Manag 2007 21 229 245 10.1108/14777260710751717 17713185

[B42] NDoH A Report on the Work Value of Nurses Employed in Public Health Facilities 2008 National Department of Health, Port Moresby

[B43] Izard J Dugue M Moving Toward a Sector Wide Approach - Papua New Guinea - The Health Sector Development Program Experience 2003 Asian Development Bank, Manila

[B44] Mandie-Filer A Bolger J Hauck V Editor ed.^eds Papua New Guinea's health sector - A review of capacity, change and performance issues Book Papua New Guinea's health sector - A review of capacity, change and performance issues 2004 ECDPM, City

[B45] WHO The World Health Report 2006: Working Together for Health 2006 World Health Organization, Geneva

[B46] Asian Development Bank Australian Agency for International Development and The World Bank: Strategic Directions for Human Development in Papua New Guinea 2007 The World Bank, Washington DC

[B47] Perry C LeMay N Rodway G Tracy A Galer J Validating a Work Group Climate Assessment Tool for improving the performance of public health organizations Hum Resour Health 2005 3 10 10.1186/1478-4491-3-10 16223447 PMC1276808

[B48] Bartram T Joiner TA Stanton P Factors affecting the job stress and job satisfaction of Australian nurses: implications for recruitment and retention Contemp Nurse 2004 17 293 304 10.5172/conu.17.3.293 15551680

[B49] Sellgren SF Ekvall G Tomson G Leadership behaviour of nurse managers in relation to job satisfaction and work climate J Nurs Manag 2008 16 578 587 10.1111/j.1365-2934.2007.00837.x 18558928

[B50] Adams A Bond S Hospital nurses' job satisfaction, individual and organizational characteristics J Adv Nurs 2000 32 536 543 10.1046/j.1365-2648.2000.01513.x 11012794

[B51] Hegney D Plank A Parker V Extrinsic and intrinsic work values: their impact on job satisfaction in nursing J Nurs Manag 2006 14 271 281 10.1111/j.1365-2934.2006.00618.x 16629841

[B52] Hagopian A Zuyderduin A Kyobutungi N Yumkella F Job satisfaction and morale in the Ugandan health workforce Health Aff (Millwood) 2009 28 w863 w875 10.1377/hlthaff.28.5.w863 19661112

[B53] Dieleman M Toonen J Toure H Martineau T The match between motivation and performance management of health sector workers in Mali Hum Resour Health 2006 4 2 10.1186/1478-4491-4-2 16469107 PMC1402315

[B54] Government of Papua New Guinea National Health Plan 2011–2020: Vol. 1 2010 Policies and Strategies, Port Moresby

